# Report of the Special Committee on Diseases of the Eye

**Published:** 1863-07

**Authors:** E. L. Holmes

**Affiliations:** Chicago; One of the Surgeons of the Chicago Charitable Eye and Ear Infirmary


ARTICLE XVI.

### REPORT OF THE SPECIAL COMMITTEE ON DISEASES OF THE EYE.

By E. L. HOLMES, M.D., of Chicago. One of the Surgeons of the Chicago Charitable Eye and Ear Infirmary.

Read at the Annual Meeting of the Illinois State Medical Society, held at Jacksonville, May, 1863.

In offering this Report, your Committee on Diseases of the Eye cannot but deeply regret that the members of the Society, generally, have not manifested more interest in the important subjects for which the Special Committee was appointed. The attention of the profession was called to the subject by public and private notices, with the request, that members should contribute whatever they could of interest relating to diseases of the eye.

Your committee has received no communications from a single member, although, many friends of the Society must have had extensive experience in ophthalmic diseases, and could contribute valuable and interesting papers upon the subject. It should not be forgotten, however, that many of our most active members have devoted their whole time and energies to the care of the brave soldiers which the State has sent into the field during the past two years. The Committee has, consequently, been left alone; and the following Report must, necessarily, be much more limited in scope than the importance of the subject merits.

Among the subjects in which we have for some time been particularly interested, and, in discussing which, we have sought the aid of the members of the Society and of the profession at large, we would mention "The Causes of Conjunctivitis in this State and North-West," and the "Prevention and Treatment of Sympathetic Ophthalmitis." Regarding the former of these subjects, we have comparatively little to say, since few facts have come to our knowledge in addition to those reported by your committee at the last meeting of the Society. Careful enquiry concerning the history of a very large number of patients, afflicted with severe conjunctivitis or its sequelæ, together with conversation with physicians practicing in different portions of the North-West, have confirmed the opinions expressed in our last Report,—that the chief causes of catarrhal ophthalmia, in this portion of our country, are found in the dry condition of the atmosphere, the bright light of the sun, rendered, possibly, more intense by this dryness, in the winds loaded with dust, and sweeping over the unbroken level of the country, and, especially, in the reckless manner in which the people expose themselves to the active causes of this disease. Several of these cases are, undoubtedly, the same which have always rendered the people of Egypt and Syria liable to conjunctival inflammation. In every portion of the world, we believe, where the climate and geological peculiarities of the country are similar to those of the North-Western States, the same diseases are prevalent. There is a popular opinion that the dust from the leaves of certain trees and flowers is one of the active causes of the disease in question. That the above are the chief causes, may be inferred from the fact, that the majority of patients examined were first attacked in the summer, when so much more exposed to these influences, than in colder seasons of the year. It is worthy of notice, that in Chicago and other places near the lake, where the atmosphere is much more moist than in those situated at a distance from it, inflammatory diseases of the conjunctiva are comparatively rare. We have been informed by intelligent physicians, practicing in the southern and western portions of this State, that severe epidemics of this disease are quite prevalent in some seasons, nearly one-half of the cases which they are called upon to treat, being conjunctival diseases of the eye. During the past seven years, we have met with no epidemic conjunctivitis in Chicago.

One of the most obvious causes of conjunctivitis is contagion. To this cause may be traced the rapid spread of this disease among the soldiers of the European armies. As soon as proper means were adopted to seclude the infected soldiers, the disease was speedily eradicated. We have been informed by physicians practicing in different portions of Illinois and the North-West, that the manner in which the small dwellings of the people are often crowded with occupants, their careless habits, as regards cleanliness, tend to increase the number of victims of this troublesome disease. We have known repeated instances in which an individual was attacked with muco-purulent conjunctivitis, and, in the space of a few weeks, the whole family, varying, in number, from four to six members, became affected. There could be no doubt that the disease was communicated, in these cases, from one member to another, by the want of proper care in the use of handkerchiefs and towels.

An indirect cause of the spread of this disease is found in delay in seeking proper medical treatment, on the part of the patient, and too often in the insufficient or too severe treatment of the physician. It was our intention to make the treatment of conjunctivitis the special subject of this Report; and, although your committee have accumulated much material upon the subject, with the history of a large number of cases, we are not yet full prepared to furnish such a paper as the subject demands. At some future time, if it meets the approval of the Society, we propose to pursue this topic.

Several cases of sympathetic ophthalmitis have fallen under our notice during the past two years. The eyes, at our first examination, were found so far disorganized as to render sight irretrievably lost. Undoubtedly, every physician of experience has either treated or observed patients who have become *entirely* blind, in consequence of an injury of only one eye. It is well known that in cases of certain injuries of the globe, especially punctured wounds, a chronic inflammation of the internal tissues of the eye supervenes, which is exceedingly difficult to overcome by any kind of treatment. After a time, varying from a day to six weeks, in a certain proportion of cases, the other eye becomes affected with a peculiar form of inflammation, principally involving the *iris*, *choroid*, *and retina*. This inflammation, at first, is usually quite slow in its progress, and is attended with pain in and around the eye with loss of sight. Regarding the serious character of this disease, MACKENZIE says: "Whenever I see sympathetic ophthalmitis, even in its first stage, I know I have to contend with an affection which, however slight its present symptoms may be, is one of the most dangerous inflammations to which the organ of vision is exposed. I have very seldom seen an eye recover from sympathetic ophthalmitis."

The curative treatment of this disease, which is now most successful, is said to be the removal of a piece of the iris, as now so highly recommended in glaucoma. We have never had an opportunity of trying it, and can, therefore, say nothing from personal experience. The preventive treatment of sympathetic ophthalmitis, which has been found, by experience, most reliable, consists in the partial or total extirpation of the injured eye, before the least symptoms of inflammation has appeared in the other. For favorable notices of this operation, we would refer to various medical journals, especially *London Ophthalmic Hospital Reports*, and *Archive of Ophthalmology*.

There are three classes of cases, according to GRÆFE, in which sympathetic ophthalmitis is especially liable to occur.—1st.—When a foreign body or a dislocated and enlarged lens is a source of irritation in one eye. 2d.—When internal disorganization is progressing, with increased outward pressure of the fluids of the eye, rendering the walls of the globe more tense than natural. 3d.—When, in commencing atrophy of one eye, (from chronic inflammations of iris and choroid,) there is tenderness, on pressure, over the region of the ciliary processes. It is impossible, however, to foretell in what cases the sympathetic inflammation will or will not make its appearance. Many patients escape after long attacks of disorganizing inflammation; but we believe it is safer, in *all* cases where vision is destroyed, to resort to the partial or total extirpation of the eye, before the symptoms of disease appear in the other eye.

We think, any one who has had much experience in treating injuries of the eye, must be fully convinced that many patients, with penetrating wounds of the cornea and lens, attended with prolapsus and inflammation of the iris and choroid, and with loss of vision, would escape much distress by resorting immediately to the surgical treatment mentioned above. Patients, however, almost invariably feel great repugnance at the "thought of having an eye cut out," and will seldom submit to an operation, till worn out with excruciating pain, and loss of sleep, and threatening loss of health.

The method of removing the eye, as introduced by Critchett, and now recommended by many ophthalmic surgeons, is simple and, usually, without danger.—A circular incision is made through the conjunctiva, parallel to the cornea, and the recti-muscles severed close to the sclerotica, as in the operation for strabismus; the conjunctiva is then separated from the sclerotica, and the optic-nerve divided by a pair of blunt-pointed curved scissors passed behind the globe, when the eye can be readily removed from the orbit and the operation finished by cutting the oblique muscles. It is, occasionally, advisable to enlarge the palpebral fissure by slitting the tissues at the external angle, especially when the eye is hypertrophied. The operation, as thus performed, leaves the muscles and nearly the whole conjunctiva. The wound usually heals in about a fortnight, leaving a "stump" suitable for an artificial eye. In many instances, the excision of the cornea is sufficient. It is not, however, always reliable, for the inflamed choroid and adjacent membranes may continue a source of irritation, which will be difficult to relieve. Moreover, the operation is not unfrequently followed by serious hemorrhage, since the inflammation has caused an enlargement of the vessels of the retina and choroid. The walls of these vessels, especially those of the retina, are exceedingly delicate, and often rupture "spontaneously" when the pressure of the humors within the globe is removed, as, in excising the cornea. The operation for the extirpation of the eye is almost entirely free from this objection, as it is seldom attended with much loss of blood. We dwell somewhat at length upon this point, because many physicians seem ignorant of the terrible consequences liable to follow certain inflammations of one eye. We are confident sight could have been preserved in six cases we have noticed, by the operation above indicated, where, by its omission, total blindness was the final result. In three cases of injury, where we had advised the operation and the patients had disregarded our council, they returned, after three or four months of excruciating pain and vain efforts to obtain relief, with general health much impaired by suffering and loss of sleep, and requested us to do as we deemed best.

It is impossible for your committee, within the limits of this Report, to present a detailed account of all the advances which have been made during the past few years in ophthalmic science. We must, however, call the attention to a few points which are of particular importance, referring, for their full discussion, to the original articles of those writers, who are regarded as high authority. The subjects of Glaucoma and the Ophthalmoscope are each worthy of a paper more extended than this Report. The medical literature of our own language is now quite rich in translations from foreign languages or in original contributions on these subjects; and we can, for the present, only refer the members of the Society to them.

A modification in the operation for the extirpation of hard cataract, ably discussed in a recent pamphlet by MOOREN, will reduce the danger attending this delicate operation, if we may judge from reported cases and from the results of two operations performed by your committee. The modification consists in removing a small section of the iris, as in performing the operation for artificial pupil, and, a week or two after, extracting the lens in the usual way. Although the pupil will, of course, be larger than natural and of an abnormal shape, the lens escapes so readily, without undue violence to the iris, that the danger of subsequent inflammation is greatly reduced.

The whole subject of accommodation of the eye to different distances, and the diagnosis of diseased condition of this function, with the use of lenses, should be carefully reviewed by every physician. The works of DONDERS, GRÆFE, WELLS, and KNAPP deserve special study.

The subject of "Astygmatismus" or difference of convexity of the cornea or lens in their different merideans, first analyzed, we believe, thirty or forty years ago by YOUNG and AIRY, is of particular interest, and has been treated, at length, by DONDERS, KNAPP, and others.

We cannot omit a short notice of a simple operation, which will prove of great benefit in certain cases of chronic inflammation of the conjunctiva with granulations, and the secondary afflictions produced by them. In many cases of these diseases, the palprebal fissure becomes diminished in size, and the lids press unduly upon the cornea in consequence of spasmodic contractions of the orbicular muscle. The double operation consists, first, in elongating the palpebral fissure at the external commissure, by an incision of a couple of lines or more in length through the conjunctiva and integument. To prevent re-union, a couple of stitches are necessary for uniting the conjunctiva and skin at the angle of the incision. The spasm is still further reduced by the second step of the operation, which is the introduction of two or three tightly drawn vertical ligatures through a fold, embracing the integument and muscle of the lid. The ligatures ulcerate out in a week or ten days. Excellent observations upon this operation can be found in the valuable work (Part I, pages 6 and 7,) of PAGENSTECHER and SÆMISCH, of Wiesbaden, published in 1861. To Dr. E. WILLIAMS, an able and well-known oculist of Cincinnati, is due, we believe, the credit of devising, independent of GAILLARD and PAGENSTECHER, the operation above described.

Your committee take pleasure in referring to the efforts of Dr. HOMBERGER, of New York, to establish a journal of ophthalmology in the United States. Such a journal, in the present rapid advances in ophthalmic science, is a much needed acquisition to our professional literature. We believe Dr. HOMBERGER, from his knowledge of the details of ophthalmic practice as observed in Europe, and his acquaintance with the ophthalmic literature of all the important European languages, will make the journal what the wants of the profession require. We heartily commend the enterprise of Dr. HOMBERGER to the support and encouragement of this Society and of the profession at large.

We would call the attention of the Society to the continued prosperity of the "Chicago Charitable Eye and Ear Infirmary," which has now been in operation five years. The organization consists of a Board of twelve Trustees, of two Consulting and two Attending-Surgeons. During the past two years, there have been 644 patients under treatment,—making an aggregate of 1224 since the opening of the Infirmary in 1858.

The Infirmary is a charitable institution, and is intended for the poor patients of the whole North-West as well as of Chicago. In nearly every large European city, and in the larger cities of the Eastern States, infirmaries have been established for the treatment of the poor suffering from the diseases of the eye or ear; and supported not only by private munificence, but also by ample grants from the State. The sum of nearly $100,000 was raised by subscription in the Cities of New York and Boston for their respective infirmaries. "There is urgent need of a similar institution in Chicago. The city is already the most important point in the North-West in every thing that relates to commerce and wealth. Every year is rapidly adding to the population of the city as well as the influence which it is exerting upon the growing country around. The inhabitants of the North-West, more especially the poor, who are particularly exposed to all the causes of this class of diseases, are more liable to diseases of the eye than those of almost any other section of the country." The members of this Association should remember that the treatment of ophthalmic diseases is one of the most important fields of labor in which the physician can devote his energies. The medical student, especially the one who intends to practice in the country, should understand thoroughly the diagnosis and treatment of the ordinary inflammatory diseases of the eye. By no means can he well prepare himself for this branch of practice, as by regular attendance upon the clinics of a well-organized eye infirmary. Your committee would, therefore, urge upon the Society a careful consideration of the claims of the Infirmary at Chicago, as a public charity and a means of extending a knowledge of diseases of the eye and ear among medical students, and ask for it such encouragement and support as the members of the profession are able to give.

